# Challenges of peer assisted learning in online clinical skills training of ophthalmology module

**DOI:** 10.1186/s12909-021-02959-3

**Published:** 2021-10-13

**Authors:** Sumera Nisar, Usman Mahboob, Rehan Ahmed Khan, Durraiz Rehman

**Affiliations:** 1 Batterjee Medical college, Jeddah, Saudi Arabia; 2 University of Lahore, Lahore, Pakistan; 3 Institute of Health Professions Education & Research, Khyber Medical University, Peshawar, Pakistan; 4 Centre for Medical Education, University of Dundee, Dundee, UK; 5 Islamic International Medical College, Riphah International University, Islamabad, Pakistan; 6 King AbdulAziz University, King AbdulAziz Medical College, Jeddah, Saudi Arabia

**Keywords:** Peer-assisted learning, Clinical skills, Learning, Curriculum

## Abstract

**Background:**

Online communication has taken over in the last 2 years due to the frequent lockdowns because of the COVID-19 pandemic. Overburdened physicians in this pandemic are struggling to get enough time to teach clinical skills online to the students. Also, due to student’s safety issues, the students cannot fully attend the clinics. Therefore, in this scenario, online PAL (Peer Assisted Learning) sessions for clinical skill teaching and learning can be an effective alternative for undergraduate medical students. The academic limitations caused by the COVID-19 related lockdown however can have a pleasurable outcome if certain challenges, related to online PAL, are overcome. Therefore, the present study aims to identify the challenges of Peer Assisted Learning (PAL) sessions during online clinical skills training in the Ophthalmology module of undergraduate medical students.

**Methods:**

This qualitative exploratory study, utilizing online focus group discussions to explore the challenges of online PAL in training and learning of clinical skills were carried at the Ophthalmology department of Batterjee Medical College, Jeddah; Saudi Arabia. A purposive convenient sampling technique was used to collect data. Data were transcribed and analyzed using thematic analysis.

**Results:**

The study identified six themes that were further divided into smaller subthemes. The subthemes derived from the collected data were organized under the following major themes; infrastructure, learning environment, psychological problems, interaction deficit, learning desires, and desire for feedback on performance. The major challenge reported by the medical student during online PAL sessions was infrastructure in terms of network connection, scheduling, and timing of the session. The unprofessional learning environment, psychological problems in terms of behavioral issues and personality changes, interaction deficit with peers, tutor, and patient, learning desires, and desire for feedback on performance were the other important challenges faced by the students.

**Conclusion:**

The challenges explored by our study can be used by the medical educators to incorporate online PAL as an effective, efficient, and alternative teaching and learning modality in the curriculum especially in compromised circumstances like the current COVID-19 pandemic.

**Supplementary Information:**

The online version contains supplementary material available at 10.1186/s12909-021-02959-3.

## Background

The online communication has taken over for the past 2 years because of the COVID-19 pandemic with lockdown in many parts of the world. With this lockdown when most of the on-campus teaching and learning and patient communication has been ceased, online teaching and learning in undergraduate medical schools have become an important and sole alternative. Online mode is a good option to convey knowledge and assure the safety of the students but teaching online clinical skills to the medical students without interaction with the actual or simulated patients has become a great challenge.

Peer assisted learning (PAL) involves people from similar social groupings who are not professional teachers, helping each other to learn and learning themselves by doing so [1]. Learning from peers is a part of the informal curriculum of medical schools for decades with lots of benefits [2]. It gives a valuable method of enhancing student’s learning experience.

More knowledgeable peers facilitate discussions and clinical skills teaching sessions to help the transition of less knowledgeable students into more knowledgeable and skilled professionals [3]. By this practice of peer-assisted learning, acquisition of knowledge and skill takes place through active helping and support among status equal or year specific matched companions [4]. It also promotes a supportive classroom atmosphere and includes time for independent work [4]. Moreover, PAL creates an environment of collaboration and cooperation among the students as well as the faculty [5].

Learning clinical skills in the clinics is a fundamental part of medical education. But tough student schedules, non-availability of physicians in the clinics due to their hectic schedules, non-availability of rare cases in the clinics, and patient safety and privacy protocol have restricted the clinical exposure of the undergraduate medical students. Added to this, the COVID-19 pandemic led to social isolation and lockdown has made it more difficult for undergraduate medical students to attend the clinics. Overburdened physicians in this pandemic cannot get enough time to teach clinical skills online to the student and due to student’s safety issues, they cannot attend the clinics. Therefore, in this scenario, online PAL sessions on clinical skill teaching and learning can be an effective alternative for undergraduate medical students.

The limitations caused by the corona-related lockdown however can have a pleasurable outcome if certain challenges, related to online PAL, are overcome. This study will explore the challenges in conducting online PAL sessions during clinical skill training in the Ophthalmology module and will help to pave the path of online PAL as an effective and efficient entity in the undergraduate medical curriculum [6]. The improvement of the online PAL as extrapolated from our study may also lead to greater student satisfaction.

## Methods

### Study Design & Setting

A qualitative exploratory study was carried out for 7 months (December 2019 to July 2020) at Batterjee Medical College, Jeddah; Saudi Arabia to explore the challenges that 4th year undergraduate medical students faced while learning clinical skills through online PAL.

### Ethical approval

Ethical approval was granted by the Ethical Review Committee (University College of Medicine & Dentistry (UCMD), University of Lahore (UOL); Pakistan/Ethical Review Committee – Ref. # 01/20/07) and informed written consent was taken from the participants.

### Participants

Participants of the study were selected by purposive convenient sampling. Two peer tutors (one male and one female) from 4th year MBBS were selected based on their good academic grades and willingness to participate in online PAL. Two peer tutor clinical skill training sessions were conducted by the first author. Four online PAL sessions were conducted to teach clinical skills of visual acuity examination, extraocular muscle movement examination, cover uncover test, and confrontation visual field examination. After PAL sessions, two pre-planned online-focused group discussions of 2 hours each were arranged to collect the data by the first (SN) and fourth (DR) author.

In total, 22 students participated in FGDs, eleven in each. The participants were selected based on their academic grades and willingness to participate in a way that both groups of FGD include a mix of students (high achievers, average students, and low achievers, 8 males, 14 females) to gain a thick descriptive of the fourth-year medical students. The FGD participants were informed about voluntary participation and informed written consent was taken.

### Instrument development

The data collection tool for the study was Focus Group Discussion (FGD). A focus group discussion (FGD) is a conversation between a homogenous or heterogeneous small group facilitated by a moderator [7]. The conversation gives different opinions on the topic under discussion. An FGD is used in situations where the topic of discussion is of common interest to the participants and does not have the issue of disclosure of sensitive information [8]. Further, it is done when getting participants around one table is not difficult; or if the topic needs to be investigated in-depth through discussion to further explore it [9]. The sample required for FGD is small due to the qualitative nature of the data.

To develop the FGD items, steps for instrument development were followed as suggested in AMEE guide 87 [10]. Step-1 included a literature review which was done to align the online PAL with prior research. Already existing survey scales and items were also explored. Overall twelve articles were selected to be included in the literature review to align online PAL with the prior research. There was limited data available on online PAL but many other supporting studies were found related to the use of PAL in clinical skill learning.

Step-2 was a short FGD of 20 min that was conducted with the 4th year students to learn that how they describe and conceptualize the online PAL. Steps 3 & 4 included the analysis of the FGD data to develop the open-ended questions. Step 5 included expert validation of the items that were done by sharing expert validation forms with four experts, two from basic science and two from the clinical science department to get a holistic perspective. The response received was discussed and analyzed to finalize the items. Step 6 included pilot testing the FGD questions for credibility and reproducibility.

### Data collection and analysis

The proceedings of the discussions were audio-recorded. Open-ended questions were asked from the participants about the challenges they faced in online PAL sessions while learning clinical skills. Data collected from the FGDs were recorded and transcribed for analysis by the first (SN) and fourth (DR) authors. Thematic analysis was carried out initially by the first author (SN) to find out the patterns within the data and to generate themes. Initial familiarization with data was done and codes identification done by open coding process, which means, “working intensively with data line by line, identifying themes and categories that seem of interest” [11] and then elucidated themes from the connection of these classifications (selective coding) [11]. Finally, by the merging of the open and axial codes, sub-categories (subthemes) were made and arranged under a central theme. This initial data analysis was shared with UM and DR for further discussion. Subsequently, summaries of the transcripts and identified themes were discussed and reviewed with the UM and DR to clarify. UM and DR reviewed the analyzed data and authors 1, 2 and 4 developed a consensus over the final analysis during an online meeting.

## Results

The objective of the study was to explore and identify the challenges encountered by medical students in learning clinical skills through online PAL sessions. Our study has explored the challenges faced by the 4th year undergraduate medical students during online PAL and discussed and shared their views in online focus group discussions. The discussion in FGD started with an initial opening question: *What did you learn today in an online clinical skills PAL session?*

Most of the students were well aware of informal PAL but they found formal online PAL as a new and exciting experience. They made many positive comments about learning from their peer and considered it a better way of application of theoretical knowledge in a fearless and supportive environment. They also found it a safe way of communication and learning during these special circumstances of the COVID 19 pandemic.

Although, majority of the students valued this new instructional tool for their learning especially when they were not able to attend the clinics due to COVID 19 pandemic-related lockdown still they had many reservations. The result of our study also helped to find out the challenges that the students encountered during the online PAL sessions. Data analysis identified six main themes with relevant subthemes mentioned in Table 1.

**Table 1:** Challenges of online PAL in clinical skill training

#	Theme	Sub-theme	Representative quotes	Solutions
1	Infrastructure	• Network connection • Scheduling • Timing/duration of sessions	“Internet connection is a big issue all the time.” “I feel that time for the session was too short to learn clinical skills by a big batch of students.”	Improve network connection More PAL sessions can be added Timing of the PAL sessions can be increased for student’s practice
2	Psychological issues	• Behavioral • Personality • Distrust on peer tutor • Skill deficit (use of gadgets)	“I found poor motivation to start work and learn coz of no direct contact with the teacher.” “I felt little confused whether I am getting right skill or not while learning clinical skills from my class fellow.” “I think that our tutor needed more training because in the question-answer session he could not give satisfactory answers to my question.”	Motivational discussion sessions with the tutor sum-up sessions by the physician (tutor) himself More meticulous training sessions for the peer tutors
3	Learning environment	• Professionalism • Acceptability (culture of safety)	“I do not feel myself in a professional environment that’s why get distracted most of the time.”	Meticulous questioning on different steps of clinical skill by the tutor
4	Interaction deficit	• Patient • Physician	“No direct contact with the patient.” “Honestly, I prefer live and personal interaction with the doctor and watching and learning from her directly in clinics especially in clinical subjects like ophthalmology. It is impossible to compare it with attending clinics or rotations.”	Videos of actual performance of tutor on his patients Add multiple videos of the skill performances In-camera skill performance by the physician
5	Learning preferences	• Desire for supervision • Desire for training	“I prefer the session to be conducted by the doctor as we gain experience from the doctor which of course more skilled and well versed.” “No hands-on experience which I think is very important in learning clinical skills.”	Videos of the actual performance of tutor on his patients
6	feedback on performance		“Feedback on performance, I missed it. Due to lack of practice, I feel that I can still make many mistakes while performing this clinical skill and nobody would correct me or would be there to give me feedback on my performance (whether I am right or wrong).”	Feedback session by the tutor himself at the end of each session

The learning environment, interaction deficit, and learning preference were the most arduous challenges faced by the students while learning clinical skills through online PAL. Lack of patient and physician interaction, their desire for physician supervision, and his feedback on performance made online PAL more challenging.

## Discussion

The world, these days, is facing one of the biggest public health crises globally due to COVID-19. The infectivity and fatality of the disease have had a significant socio-economic, political and psychological impact on human life. Millions of people have been quarantined at their homes due to global lockdown to implement social distancing measures to avoid infectivity and control the spread of the virus [12]. This social isolation has been compounded by panic, stress, and anxiety and has been crucially affecting human mind. The health system including medical students is under great amounts of stress.

Our study revealed the importance and need of online PAL in the current lockdown situation related to the COVID-19 pandemic. The pandemic related global lockdown has led academic activities online in many parts of the world due to the redundant spread of virus infectivity and student safety. Due to this sudden shift of teaching strategy, teachers have become over occupied in making their lectures and other academic activities compatible with new learning requirements. Meanwhile in clinics, due to the increased patient load in hospitals, the physicians are struggling to teach clinical skills to medical students. In such a scenario, online PAL has emerged as a useful alternative instruction tool to train medical students about clinical skills.

At the same time where online PAL is a highly needed and desired instructional tool, it also has its challenges [13]. With the desire to make online PAL an effective instructional tool in the future, we have explored the challenges of online PAL while teaching clinical skills to medical students. Also, the literature review found many supporting studies related to the use of PAL in clinical skill learning. Joanna Hong Meng Tai emphasized the successful use of PAL in clinical skill learning to address the challenges of the real world [14]. Diane O’Doherty discussed the importance of the use of online learning in medical education. Medical educators are already under pressure to find sufficient time to manage to reach, research and maintain a work-life balance personal life commitment. Online learning can be a useful emerging modality in medical education in such conditions [15]. Anita Raymond talked about the significance of online PAL as an effective way to teach and learn which promotes convenient and flexible learning. Peer assisted online learning groups helped to reduce the social isolation of the shy students and helped in fearless communication [16].

Inadequate infrastructure deployment can be an eminent cause of online learning failure. We have also found that infrastructure-related problems were one of the main concerns of the students. Although, most of the students agreed upon the importance and demand of online PAL in the current situation of COVID-19 related lockdown, at the same time they indicated some important problems they faced while their online learning. A strong internet connection is the first step in conducting any successful online session [17]. A poor internet connection can affect learning in many ways. It can affect the quality of videos and sound of the session, causes a delay in communication with a peer tutor, and problems related to downloading the videos or recording of the sessions. Many of them found difficulties in logging in to the system which caused them the stress of being left behind than their class fellows. Few found them absent on the system while they were still in the session [18]. They also found that the timing of each session was not sufficient to learn clinical skills. Daroedono has already mentioned the importance of enough timing in learning clinical skills in his study [19]. Clinical skill learning is a complex task that needs enough time to learn and practice [20].

In classroom settings, the teacher can employ different ways to manage a student’s day-to-day behavior, emotional challenges, and interpersonal skills to help them to learn. During lockdown related online learning, especially online PAL, which is taken by their peers, our study has discovered many psychological challenges experienced by the medical students [19]. Lack of motivation to learn was the substantial problem faced by many students caused chiefly due to a lack of interaction with the physician and patient. Owusu-Fordjourhas also explored that demotivation during online learning caused many students additional lethargy, coupled with an additionally sedentary lifestyle due to the corona-related lockdown [21]. Uncertainty about the future has created stress and anxiety among the students which led them into the habit of delaying their study time. This poor time management indirectly affected their work efficacy and ability to meet deadlines [22].

Distrust in peer tutors made them skeptical about peer tutor’s knowledge and skill expertise and wanted to learn skills directly from the expert (physician) [23]. Despite their liking of online PAL in current special circumstances and encouraging positive comments from the students, it was apparent that most of them wanted direct contact and direct learning from the expert [24]. Direct contact with the expert (physicians) in clinical settings and time spent with them was more effective and valued by the students than learning the content. Being in their company and connectivity with them in clinics fulfills many purposes [25]. This also fulfills their desire to be supervised in their learning of clinical skills. This desire to be supervised or to be in direct contact with the expert or physician has made online PAL more challenging.

Well-planned online learning is usually meaningful but, in the current unpredictable situation of crisis management of teaching and learning, most of the online sessions were put in place as an emergency measure to cope with the situation [26]. Psychologically stressed students also need proper training to learn to yield the maximum benefit in these special circumstances [27].

Feedback on performance is essential in clinical education and has a powerful influence on learning [28]. Observation of the physical performance of others and positive comments contribute to learning. In clinical settings, comments on skill performance by the physician or expert are the most desired response for the students to enhance their performance and understanding [29]. Constructive feedback on performance always encourages and motivates the students to learn [30]. Lack of constructive feedback on performance from the physician (expert) himself was another deficiency felt by the participants of our study. They found themselves confused or clueless about the success of their learning.

### Limitation of the study

Due to unexpected and unprecedented COVID-19 related lockdown, we felt that having more time to prepare or train the peer tutor for online sessions would have further improved the student’s experience of online PAL.

## Conclusion

While being in the midst of the COVID-19 crisis, the medical academic community must learn from their experience and prioritize to draw practical solutions to train medical students in these unprecedented times. Within the constraints of the current healthcare situation, integration of online PAL into the curriculum from the outset would help the medical students to become self-accountable for their learning. By overcoming the challenges explored by the study, online PAL can be an effective alternative for teaching and learning not only for academic knowledge but also for clinical skill training. It creates a successful virtual learning environment for students to practice clinical examinations or history taking among themselves with the help of a more knowledgeable peer tutor in a comfortable non-threatening environment. The use of a wide variety of online resources will also help the students to promote their learning. Its integration in the curriculum to train medical students for clinical skills may reduce the need for a basic clinical educator, especially in such special unprecedented circumstances when they are increasingly busy.

## Supplementary Information

**Additional file 1.** Supplementary data. Annexes containing. FGD items. Consent form for students. Content validation form.

## Data Availability

The datasets used and/or analyzed during the current study are available from the corresponding author on reasonable request.

## Abbreviations

PAL: Peer Assisted Learning
FGD: Focus group discussion
BMC: Batterjee Medical College
UOL: University of Lahore
