# Supplementary material for: Challenges of peer assisted learning in online clinical skills training of ophthalmology module
Source: BMC Med Educ. 2021 Oct 13;21:530. doi: 10.1186/s12909-021-02959-3 (PMC8512649; doi:10.1186/s12909-021-02959-3)
Supplement: Supplementary file 1 — Additional file 1. Supplementary data. Annexes containing. FGD items. Consent form for students. Content validation form. [file 12909_2021_2959_MOESM1_ESM.pdf]

Supplementary data

Challenges of Peer Assisted Learning in online clinical skills training of Ophthalmology module

Sumera Nisar[a], Usman Mahboob[b], Rehan Ahmed Khan[c], Durraiz Rehman[d]

[a]Batterjee Medical college, Jeddah, KSA, [b]Institute of Health Professions Education & Research, Khyber Medical University, Peshawar, Pakistan, Associate, Centre for Medical Education, University of Dundee, UK, [c]Islamic International Medical College, Riphah International university, Islamabad, Pakistan, [d]King AbdulAziz University, Jeddah, KSA

[a]Dr. Sumera Nisar MBBS FCPS
Professor in Ophthalmology
Batterjee Medical College, Jeddah
KSA
[b]Dr. Usman Mahboob MBBS, MPH, DHPE, FHEA, Fellow FAIMER
Assistant Professor in Medical Education
Institute of Health Professions Education & Research
Khyber Medical University
Peshawar, Pakistan
Associate, Centre for Medical Education
University of Dundee, UK
[c]Dr. Rehan Ahmad Khan
Professor in Surgery
Riphah International University
Islamabad, Pakistan
[d]Dr. Durraiz Rehman MBBS, FCPS
King AbdulAziz Medical College, Jeddah
KSA

Annexure: 1 <u>Consent form for students</u>

<u>Title:</u> Challenges of Peer Assisted Learning (PAL) in online clinical skills training of Ophthalmology module

Dear student

You are being asked to voluntarily participate in this research study. The purpose of the study is to understand your views about PAL and its challenges while learning online clinical skills. Being 4th year medical student of BMC, you are eligible to participate in this research project.

If you agree to participate, your participation will be involved in online FGD. It will take no more than two hours. You are expected to participate in discussion during the session of online FGD. Your name will not be used during Focus group discussion and no identifying information is being collected as part of this FDG recording. All points during FGD may be answered by you according to ground rules of FDG. The proceedings of the online FGD will be auto recorded by the system. You may choose not to participate in FDG. Whether you participate or not will not affect your education. There are no known risks from your participation. No direct benefit from your participation is expected. There is no cost to you except for your time. You will not be paid for participation in this study. Only the study team will have access to the information that you provide, which will remain anonymous. Data from all respondents will be transcribed and then summarized in themes.

Completing this survey indicates your voluntary agreement to participate. By participating in the discussion, you are giving permission for the investigator to use your information for research purposes.

In case of any inquiry you can contact me anytime on this email address:
sumeranisr16@gmail.com.

Thank you.

Annexure: 2

<u>FGD items</u>

Q1. What did you learn today in online clinical skills PAL session?

Q2. How is your experience of online clinical skills PAL session?

Q3. How does it add differently to your learning as compared to other live teaching strategies?

Q4. What are the challenges or difficulties you faced for the online clinical skills PAL session? (students related, peer-tutor related, facilitator related, environment related, internet connection related, training related, lack on hands on practice related, scheduling and timing related, time-tabling related, feedback on performance related, format of session related)

Q5. How these difficulties or challenges can be improved?

Q6. Would you like to add anything else related to online PAL or its challenges?

Q7. How is this lockdown affecting your studies?

Q8. What else could have been done to facilitate your learning in the current situation of lockdown?

Annexure: 3

Content validation form

Please choose (√) the option which suits you best

Extremely unclear = 1 Somewhat unclear = 2 Neutral =3 slightly clear =4 Very clear=5

Sr. #	FGD items	1	2	3	4	5
1	**What do you know about Peer Assisted Learning (PAL)?**					
2	**Did you ever participate in any PAL session before?**					
3	**Do you have any previous experience with online teaching and learning?**					
4	**What did you learn today in online clinical skills PAL session?**					
5	**How is your experience of online clinical skills PAL session?**					
6	**How does it add differently to your learning as compared to other live teaching strategies?**					
7	**What are the challenges or difficulties you faced for the online clinical skills PAL session?** (students related, peer-tutor related, facilitator related, environment related, internet connection related, training related, lack on hands on practice related, scheduling and timing related, time-tabling related, feedback on performance related, format of session related)					
8	**Which challenge affected your learning the most?**					

9	**How these difficulties or challenges can be improved?**					
10	**Would you like to add anything else related to online PAL or its challenges?**					
11	**How is this lockdown affecting your studies?**					
12	**What did you learn in this lockdown?**					
13	**What else could have been done to facilitate your learning in the current situation of lockdown?**					

Comments:
